# Perceived exertion reflects fatigue conditions during power-aimed resistance training

**DOI:** 10.1055/a-2545-5403

**Published:** 2025-03-25

**Authors:** Hanye Zhao, Takanori Kurokawa, Masayoshi Tajima, Zijian Liu, Junichi Okada

**Affiliations:** 1 13148 Faculty of Sport Sciences, Waseda Daigaku, Tokorozawa, Japan; 2 13148 Graduate School of Sport Sciences, Waseda Daigaku, Tokorozawa, Japan

**Keywords:** borg scale, neuromuscular fatigue, resistance training, surface electromyography

## Abstract

Fatigue is an inevitable part of resistance training, making its monitoring crucial to prevent performance decline. This study evaluated the validity of ratings of perceived exertion as a measure of fatigue during power bench press exercises. Fourteen sub-elite male athletes completed three bench press tasks with varying volumes (low, medium, and high) at 65% of their one-repetition maximum. The rating of perceived exertion, a spectral fatigue index, and velocity loss were measured across all conditions. Significant effects were observed for the overall ratings of perceived exertion, average velocity loss, and average spectral fatigue index (all *p* <0.001). As tasks progressed, the rating of perceived exertion and the spectral fatigue index increased significantly ( *p* <0.001), while the velocity loss was not significant under the low-volume condition. Significant correlations were found between the rating of perceived exertion and the spectral fatigue index ( *r* =0.547, *p* <0.001), the velocity loss and the spectral fatigue index ( *r* =0.603, *p* <0.001), and the rating of perceived exertion and the velocity loss ( *r* =0.667, *p* <0.001). The findings suggest that both the rating of perceived exertion and the velocity loss are valid measures of fatigue in power bench press exercises. However, the rating of perceived exertion is a more practical option due to its simplicity and accessibility. Furthermore, the rating of perceived exertion can act as a substitute for velocity when measurement tools are unavailable. It should be noted that velocity alone may not fully capture fatigue in low-repetition power training.

## Introduction

Fatigue is a common physiological phenomenon that occurs during physical activities such as labor and sports. In resistance exercises, fatigue is inevitable 1 and leads to a reduced power output, including decreases in peak velocity and power output, due to impaired force production 2 3 . Furthermore, fatigue has been associated with an increased risk of acute injuries caused by deteriorations in exercise techniques and 4 , in some cases, a higher likelihood of developing chronic pain 5 6 . Thus, assessing fatigue is essential for optimizing athletic performance, ensuring safety, and reducing injury risks during resistance training.

Fatigue involves a range of physiological responses throughout the neuromuscular system, from central to peripheral regions. It can be assessed using various measures, such as blood lactate levels and maximal force output 2 7 8 . Surface electromyography (sEMG) is a widely used non-invasive tool for evaluating neuromuscular functions in research, sports science, and clinical applications 9 10 . sEMG signals can detect and quantify fatigue during isometric contractions 10 11 . Extensive research over the past century has established sEMG as a reliable method for identifying fatigue 10 . Fatigue-related changes in muscle fiber conduction velocity cause shifts in the power spectral density toward lower frequencies, which can be observed in sEMG signals 12 . As a result, the mean and median frequencies of the power spectrum are widely used as key indicators of fatigue during isometric contractions 10 11 13 .

Accurately assessing fatigue is essential for athletes, coaches, and physical therapists. While the use of sEMG for fatigue assessments is growing, particularly in resistance exercises 11 14 15 , its high cost and the expertise required for the proper use limit its accessibility. Even if sEMG devices were more affordable, their application requires advanced skills, particularly in processing unstable signals during resistance exercises, which makes them less practical for the widespread or individual use outside clinical and research settings 10 13 .

Timely feedback on fatigue is critical during power-aimed resistance exercises; yet, power spectrum changes occur over a relatively longer timescale. In power-aimed training scenarios (e.g., peak power and velocity), athletes must lift weights quickly while minimizing velocity decreases from fatigue 2 16 . sEMG-based fatigue assessments are impractical in these settings as they cannot provide the immediate feedback required during resistance exercises. To make fatigue assessments more practical, several indirect indicators have been developed 2 17 18 . For example, a blood lactate concentration is considered a significant indirect marker of fatigue 19 . However, its measurement typically requires invasive methods like skin puncture, causing discomfort and additional burden to participants. Moreover, blood lactate levels are easily affected by factors such as relative intensity and rest intervals during resistance exercises 20 21 , making it difficult to control for these variables. Consequently, assessing fatigue using blood lactate levels is only suitable in specific scenarios.

In the past decade, velocity-based training has gained popularity in resistance exercises, with studies showing its positive impact on athletic performance 22 23 . Affordable devices using rotary encoders or laser speedometers to measure lifting velocity have become widely available 24 25 . Velocity loss is recently recognized as a reliable fatigue indicator, correlating significantly with metabolic parameters like blood lactate and ammonia concentrations during power-aimed resistance exercises 2 26 27 . However, high-precision velocity monitoring devices remain expensive for most users. Additionally, explosive lifting may not be suitable for inexperienced individuals due to the limited muscle strength, joint stability, and proper technique 28 . It is also unsuitable for resistance training programs focused on rehabilitation or hypertrophy 29 . Consequently, the velocity-based fatigue assessment is primarily applicable to experienced lifters in power-aimed training, limiting its practicality for general practitioners.

The rating of perceived exertion (RPE) scale is a subjective, perception-based tool for quantifying exercise intensity 30 31 . Initially developed for clinical settings, it has been widely adopted in sports science due to its simplicity and reliability in reflecting physiological responses, such as heart rate, muscle activation, and power output during endurance exercises 32 33 . Over the past two decades, the RPE has gained popularity in resistance training, integrating key factors like %1RM, training volume, and rest intervals 21 31 34 . Consequently, it is increasingly used as a physiological marker in resistance exercises. Despite variations between scales (e.g., OMNI and Borg’s scales), most RPE scales are validated and reliable for quantifying physiological responses in resistance exercises. The RPE has also been found to correlate with fatigue during non-explosive resistance exercises 14 35 36 , suggesting its potential as a marker for fatigue in power-aimed exercises. However, the relationship between the RPE and fatigue in power-aimed resistance exercises remains unclear as sEMG-based fatigue assessments in these contexts are inconsistent 10 13 . While the RPE-based fatigue assessment has been validated in isometric and non-explosive exercises 14 15 37 , further research is needed to confirm its utility as a versatile tool for assessing fatigue in various settings.

Recent advancements in mathematical-based power spectrum analysis techniques have enabled the potential for quantifying fatigue during dynamic contractions 11 13 . These methods may facilitate sEMG-based fatigue assessments in explosive movements, paving the way to establish a relationship between the RPE and fatigue. Such validation could position the RPE as an accessible and effective tool for indirect fatigue assessments in power-aimed resistance exercises, benefiting practitioners in sports, physiology, and clinical fields. This study aims to (1) verify the relationship between the RPE and a new sEMG-based algorithm during power bench press (BP) exercises and (2) compare the RPE with velocity loss to determine a more practical fatigue indicator for use in power-aimed resistance training programs.

## Materials and Methods

### Experimental designs

This study investigated the validity of the RPE as a simplified fatigue indicator and compared it with velocity loss in power BP exercises. A counterbalanced crossover design was used, with BP selected due to its popularity in resistance training. The protocol included two sessions, separated by at least 48 hours. In the initial session, participants’ descriptive data were collected, and they were instructed on using the RPE scale. An anchoring trial was conducted, where participants performed a single set of power BP to physical failure, establishing the upper and lower RPE limits and determining the required repetitions for the experimental session 14 21 35 . In the second session, participants completed three conditions corresponding to 30% (L), 60% (M), and 90% (H) of their repetitions from the anchoring trial. These conditions were performed in a counterbalanced order. sEMG signals, RPE scores, and velocity data were recorded across all conditions for analysis.

### Participants

The sample size was determined using statistical power analysis software (G*Power 3.1, Bonn University, Germany). An analysis of variance (ANOVA) model with fixed effects, main effects, and interaction analyses was used. The input parameters were set at an effect size of 0.4, an alpha level of 0.05, and a power of 0.95 14 15 . The minimum required number of participants was calculated to be 14. Fifteen sub-elite collegiate athletes were recruited for the study; however, one participant failed to complete the experimental protocol, resulting in a final sample of 14 participants. The participants were active collegiate athletes who regularly engaged in daily resistance training and frequently competed at national and regional levels. All participants were ranked between the Division II league (approximately equivalent to National Collegiate Athletic Association Division II) and the regional finals in Japanese collegiate athletics. Their competition experience averaged 10.5±3.3 years. None of the participants had any neuromuscular or skeletal injuries, nor were they undergoing medical treatment. All participants were well-experienced in resistance training, with an average of 5.0±2.3 years of experience. The descriptive characteristics of the participants were as follows (mean±standard deviation): age, 20.5±1.1 years; body mass, 73.3±12.9 kg; height, 172.3±5.7 cm; body fat percentage, 15.7±4.1%; and 1RM for BP, 92.3±17.5 kg. Before the experiment, all participants were informed about the experimental schedule, measurement items, potential risks, discomforts, and benefits. Written informed consent was obtained from all participants. The study was conducted in accordance with the ethical guidelines outlined in the Declaration of Helsinki and was approved by the Human Ethics Committee of an institution affiliated with one of the authors.

### Initial session

Before the session, participants received detailed instructions on the study’s purpose, measurements, and potential risks and benefits. Written consent was then obtained. Descriptive characteristics, including body fat percentage (measured using a bioelectrical impedance machine, InBody 970, InBody Co., Ltd, South Korea), were recorded. Participants completed a warm-up consisting of 5 minutes of jogging, static and dynamic stretching, and two sets of power BP with 20 and 30 kg for eight and six repetitions, respectively. The rest interval was determined by the participants, and the next set was performed when they indicated that they were ready. This protocol has been shown to enhance power 2 38 . The participants’ 1RM for BP was measured using National Strength and Conditioning Association guidelines with a standard Olympic barbell 28 . The 1RM test involved progressively increasing the load until the participant achieved their maximum weight for one repetition.

After a 5-minute rest, participants were instructed on Borg’s CR-10 scale (0–10), which measures perceived exertion in terms of effort, discomfort, and fatigue in the upper body 21 30 . The lifting cadence was standardized with a 2-second lowering phase (eccentric) and an explosive pushing phase (concentric), controlled using a smartphone metronome (one beep/s) 14 . The safety bar was set just above the chest height to prevent barbell rebound and any potential impact on the sEMG data. Participants then practiced lifting with only the barbell to familiarize themselves with the cadence and technique. An anchoring trial followed, where participants performed a single set of power BP at 65% 1RM until failure, establishing RPE anchors. A score of 0 (“nothing at all”) represented a relaxed state, while 10 (“extremely strong”) corresponded to failure 26 39 . Successful repetitions required lowering the bar to the safety bar and pushing it back to the starting position.

### Experimental session

Before the experimental session, participants completed the same warm-up routine as in the initial session. Afterward, they practiced RPE reporting using Borg’s CR-10 scale, placed vertically above their heads for easy visibility. The lifting cadence was set to a 2-second lowering phase (eccentric), an explosive raising phase (concentric), and a 2-second pause between repetitions, controlled using a smartphone metronome (one beep/s). During the pause, participants reported their subjective exertion for the most recent repetition based on the RPE range established during the anchoring trial. If exertion exceeded the high anchor (10, “extremely strong”), participants could report scores above 10 30 40 .

Following the warm-up and RPE reporting practice, the three experimental conditions (L, M, and H) were performed in a counterbalanced order. The required repetitions for each condition were calculated from the anchoring trial. To reduce anticipatory effects (e.g., central governor influence) 41 42 , participants were informed of the required repetitions only during the second-to-last repetition and instructed to stop after completing the final one. The RPE was recorded after each repetition during the 2-second pause. If participants struggled to maintain the cadence, they were encouraged to follow the metronome as closely as possible. After each condition, participants reported an overall RPE for the trial. A 5-minute rest was provided between conditions.

### Surface electromyography

sEMG signals were recorded from the pectoralis major, lateral head of the triceps brachii, and anterior deltoid muscles on the dominant side. Bipolar surface electrodes (ADMEDEC Co., Ltd, Japan) were placed on each muscle following established anatomical guidelines 43 44 . Before the electrode placement, the skin was shaved, abraded, and cleaned with alcohol to reduce impedance. The signals were amplified using an active differential preamplifier and transmitted wirelessly to a device (MARQ MQ-8, Kissei-Com Tech, Nagano, Japan). Sampling occurred at 1,000 Hz, and a synchronized high-speed camera (Grasshopper GRAS-03K2C, FLIR Systems Inc., Canada) recorded a motion video. The raw sEMG signals were captured using Vital Recorder 2 (Kissei-Com Tech, Nagano, Japan), segmented into individual repetitions, and exported for analysis, focusing only on the concentric phases.

A fourth-order Butterworth bandpass filter (20–450 Hz) was applied to remove noise. Fatigue was quantified using the spectral fatigue index (SFI), a validated parameter shown to be sensitive during dynamic contractions 11 13 . A fast Fourier transformation was applied to compute the power spectrum, and spectral moments were calculated using the following equation (1) :



The SFI was determined as the ratio of spectral moments of orders −1 and 5, as shown in equation (2) :



SFI values were calculated for each repetition, normalized to the first repetition, and averaged across the three muscles to create a single parameter for statistical analysis. All calculations were performed using Matlab 2024a (Mathworks, Natick, MA, USA).

### Velocity loss calculations

Barbell velocity during the BP was measured using a high-speed video recorded at 120 frames/s from the sagittal plane. The camera was positioned 5 m from the bench, facing the participant’s left side. A marker on the barbell’s end was used to track its trajectory. Pixel-to-distance calibration was conducted with a 1-m stick placed near the barbell edge to convert pixel measurements into real-world distances 45 . The barbell’s trajectory coordinates were tracked, and the concentric phase velocity was calculated using a KineAnalyzer (Kissei-Com Tech, Nagano, Japan). The coordinates and velocities were segmented into individual repetitions and exported for analysis. The peak concentric phase velocity for each repetition was extracted with a custom Matlab code (Matlab 2024a, Mathworks, Natick, MA, USA). Intra-set velocity loss was determined by comparing each repetition’s velocity to the fastest repetition within the same conditions, calculated on a repetition-by-repetition basis 2 .

### Statistical analysis

The Shapiro–Wilk test assessed the normality of the overall RPE, average velocity loss, and average SFI for each condition. The average SFI and velocity loss were normally distributed, so one-way ANOVA with the Bonferroni correction was applied. However, the overall RPE under the M and H conditions did not meet the normality assumption, prompting the use of Friedman’s test with the Bonferroni correction for multiple comparisons.

To examine changes in the RPE, SFI, and velocity loss during the lifting process, data from specific repetitions (first, median, and last) were analyzed for each condition. Given the varying repetition numbers (L: 3.8±0.7; M: 7.2±1.3; H: 11.0±1.0), a two-way ANOVA (three conditions×three repetitions) was used to test for main and interaction effects, followed by Bonferroni-corrected post hoc tests if significant effects were detected. The sphericity assumption was verified for the ANOVA tests. If sphericity could not be assumed, the Greenhouse–Geisser correction was applied. If ε>0.75, a Huynh-Feldt correction was applied. The effect size for the ANOVA was indicated with partial *η* ^2^ and 95% confidence intervals (CIs). Partial *η* ^2^ and 95% CIs were calculated using the customized R code (R Studio 2024.12.0, Posit Software, PBC, MA, USA).

For correlation analysis, intra-set RPE, velocity loss, and SFI data were included. To avoid overlapping data, specific volume ranges were used: 0–30% for the L condition, 30–60% for the M condition, and 60–90% for the H condition 35 46 . Correlations between SFI and RPE, as well as SFI and velocity loss, were compared using Fisher’s *r* -to- *z* transformation and paired two-tailed tests. Statistical analysis was performed using SPSS 29.0 (SPSS Inc., USA), with significance set at *p* <0.05.

## Results

**Fig. 1:**
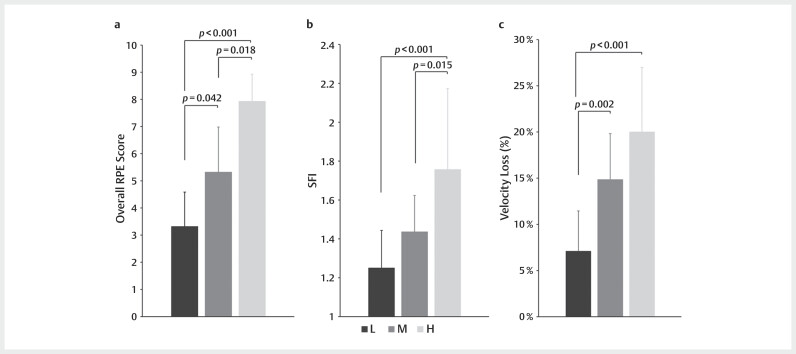
Overall RPE ( **A** ), the average SFI ( **B** ), and the average velocity loss ( **C** ) of bench press tasks. The *p* values represent the differences and significant levels between experimental conditions. RPE, ratings of perceived exertion; SFI, spectral fatigue index.

Significant differences in the overall RPE were observed across the experimental conditions ( *χ* ^2^ =27.527, *p* <0.001, and Kendall’s *W* =0.983), with specific differences noted between L vs. M ( *p* =0.042), M vs. H ( *p* =0.018), and L vs. H ( *p* <0.001) comparisons ( Fig. 1A ). For the average SFI, significant effects for conditions were observed ( *p* <0.001, partial *η* ^2^ =0.365, and 95% CI [0.115, 0.528]). Bonferroni post hoc tests indicated significant differences in L vs. H ( *p* <0.001), and M vs. H ( *p* =0.015) comparisons ( Fig. 1B ). Finally, significant effects for conditions were observed in the average velocity loss ( *p* <0.001, partial *η* ^2^ =0.496, and 95% CI [0.243, 0.632]). Bonferroni post hoc tests indicated that significant differences in the average velocity loss were observed between L vs. M ( *p* =0.002) and L vs. H ( *p* <0.001) comparisons ( Fig. 1C ).

**Fig. 2:**
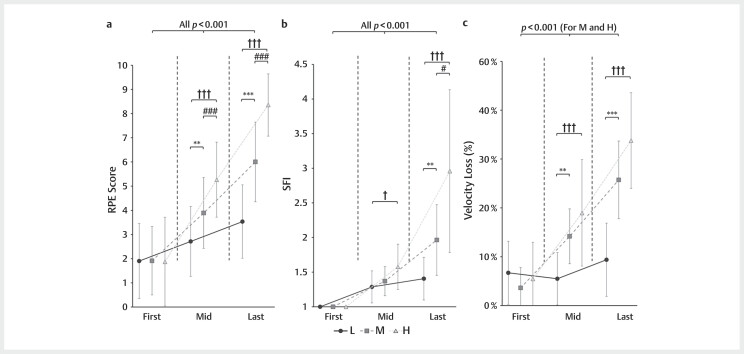
The RPE ( **A** ), spectral fatigue index (SFI) ( **B** ), and velocity loss ( **C** ) during low (L, circle with solid lines), medium (M, square with dashed lines), and high (H, triangle with dotted lines) volume conditions of exercises. *A significant difference as L compared with M conditions, ** *p* <0.01, *** *p* <0.001; ^#^ a significant difference as M compared with H conditions, # *p* <0.05, ### *p* <0.001; ^†^ a significant difference as L compared with H conditions, † *p* <0.05, ††† *p* <0.001. The *p* values indicate the differences and significant levels between selected repetitions. RPE, ratings of perceived exertion; SFI, spectral fatigue index.

Intra-set data were analyzed using a two-way ANOVA, with the first, mid-point, and last repetitions extracted for this analysis. Significant interaction effects between conditions and repetitions were observed for the RPE ( *p* <0.001, *F* =39.159, partial *η* ^2^ =0.751, and 95% CI [0.600, 0.808]), SFI ( *p* <0.001, *F* =20.744, partial *η* ^2^ =0.615, and 95% CI [0.407, 0.702]), and velocity loss ( *p* <0.001, *F* =12.029, partial *η* ^2^ =0.481, and 95% CI [0.241, 0.592]). There was also a significant overall main effect of the number of repetitions on the RPE ( *p* <0.001, *F* =77.572, partial *η* ^2^ =0.856, and 95% CI [0.712, 0.901]), velocity loss ( *p* <0.001, *F* =79.732, partial *η* ^2^ =0.860, and 95% CI [0.719, 0.904]), and SFI ( *p* <0.001, *F* =44.499, partial *η* ^2^ =0.774, and 95% CI [0.563, 0.845]) throughout the BP trials. In pairwise comparisons, significant changes over repetitions were observed under the L, M, and H conditions for both the RPE and the SFI (all *p* <0.001). However, significant velocity loss over repetitions was not observed under the L conditions ( *p* =0.238, *F* =1.517, partial *η* ^2^ =0.104; Fig 2C , circle with solid lines). Significant differences in RPE were noted between experimental conditions at the mid-point ( *p* <0.001, *F* =35.911, partial *η* ^2^ =0.734, and 95% CI [0.496, 0.818]) and last repetition ( *p* <0.001, *F* =63.952, partial *η* ^2^ =0.831, and 95% CI [0.665, 0.884]). The SFI also showed significant differences at the mid-point ( *p* =0.005, *F* =6.526, partial *η* ^2^ =0.334, and 95%CI [0.042, 0.526]), and the last repetition ( *p* <0.001, *F* =20.909, partial *η* ^2^ =0.617, and 95% CI [0.320, 0.736]). Velocity loss significantly differed at the mid-point ( *p* =0.002, *F* =11.181, partial *η* ^2^ =0.462, and 95% CI [0.141, 0.625]) and last repetition ( *p* <0.001, *F* =30.489, partial *η* ^2^ =0.701, and 95% CI [0.443, 0.795]).

**Fig. 3:**
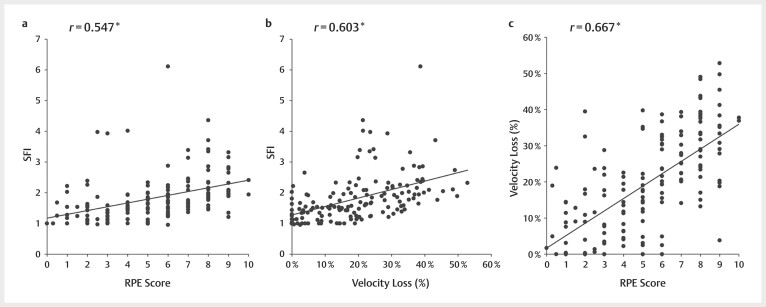
Spearman’s Rho of SFI–RPE scores ( **A** ), SFI–velocity loss ( **B** ), and RPE–velocity loss ( **C** ) during bench press tasks. *The significant level of correlation coefficients, *p* <0.001. RPE, ratings of perceived exertion; SFI, spectral fatigue index.

The results of the Spearman correlation analysis, which included a total of 154 BP repetitions, are shown in Fig. 3 . Significant relationships were found between the SFI and RPE ( *r* =0.547, *R* ^2^ =0.299, *p* <0.001; Fig. 3A ) and those between the SFI and the velocity loss ( *r* =0.603, *R* ^2^ =0.363, and *p* <0.001) were found to be significant ( Fig. 3B ). No significant difference was observed between the SFI–RPE and SFI–velocity loss correlations ( *z* =−0.723 and *p* =0.467). Additionally, a significant relationship was found between RPE and velocity loss ( *r* =0.667, *R* ^2^ =0.444, and *p* <0.001; Fig. 3C ).

## Discussion

The purpose of this study was two-fold: (1) to examine the validity of using RPE as a fatigue marker by analyzing the relationship between RPE and fatigue, quantified using a new sEMG-based algorithm during power BP exercises and (2) to compare RPE with velocity loss to determine a more practical and versatile fatigue indicator for use in power-aimed resistance exercise programs. The most important findings of this study are as follows: (1) RPE and velocity loss significantly increased with fatigue, indicating that fatigue results in an increase in RPE and a decrease in velocity during power BP exercises, (2) significant correlations were observed between the RPE and the SFI, as well as between the velocity loss and the SFI, suggesting that both RPE and velocity loss can serve as fatigue markers during power BP exercises. (3) A significant correlation between RPE and velocity loss was found, indicating that RPE could also be used as a reliable indicator for velocity loss when a velocity assessment device is unavailable during power BP exercises. However, (4) significant velocity loss was not observed under the L condition, suggesting that velocity loss may not effectively reflect fatigue when performing power training with low prescribed volume.

Subjective exertion is associated with various central and peripheral physiological responses, such as firing frequency and motor unit recruitment during resistance exercises 21 47 . Lagally et al. found that RPE, blood lactate, and muscle activation levels increased correspondingly with the relative intensity of biceps curl exercises, suggesting that RPE is linked to both central and peripheral responses 21 . To control for the influence of relative intensity, a constant relative intensity was maintained across the three experimental conditions. The significant differences in overall RPE observed in this study may be related to the differences in the number of repetitions prescribed under each condition. For instance, Hiscock et al. indicated that resistance exercise programs with higher volumes (more repetitions) elicit more severe metabolic, endocrine, and perceptual responses, even when the relative intensity is lower 18 48 . Consistent with these findings, the H condition, which had the highest volume, likely accelerated intramuscular perturbation and disruption of homeostasis (e.g., accumulation of hydrogen ions) 49 . These physiological responses increase afferent feedback from group III/IV muscle fibers, resulting in a significant rise in subjective exertion and discomfort 50 51 . Moreover, disruptions in homeostasis can affect muscle fiber conductivity, which can be quantified by analyzing the power spectral density characteristics of the sEMG signal 13 49 . Significant changes in the SFI reflect a shift in the power spectrum from high to low frequencies, a shift induced by fatigue-related mechanisms 52 . Consequently, the corresponding increases in RPE and SFI suggest that fatigue leads to similar changes in perceived exertion, indicating that RPE can effectively reflect fatigue responses during power BP exercises.

During resistance exercises, particularly in power-aimed lifting programs, it is crucial for practitioners, such as athletes and coaches, to manage fatigue levels to prevent significant velocity decreases, as highlighted in the Introduction section. With the growing trend of velocity-based training, practitioners have increasingly turned to velocity-measuring devices to monitor fatigue in power-aimed exercise programs. While these devices have become more affordable in recent years, they remain unrealistic for individuals to purchase and may not be suitable for the large-scale use. To address this challenge, we selected RPE, a widely used and nearly zero-cost alternative, as a more practical fatigue indicator for power-aimed resistance exercises. Previous studies have validated the relationship between fatigue and RPE in isometric core exercises (e.g., prone plank) 15 37 . In the present study, significant differences in the average SFI were observed between conditions (L vs. H: *p* <0.001; M vs. H: *p* =0.015). Similar significant differences were found in overall RPE (L vs. M: *p* =0.014; L vs. H: *p* <0.001; M vs. H: *p* =0.006) and average velocity loss (L vs. M: *p* =0.002; L vs. H: *p* <0.001). These results indicate that the average fatigue level, overall RPE, and average velocity loss increased correspondingly during power BP exercises. Thus, both the overall RPE and average velocity loss could serve as indicators of total fatigue of past sets or sessions. For example, practitioners could assess the overall RPE after a single set of power BP or an entire power-aimed lifting program to gauge the overall fatigue level of the lifting contents. Similarly, they could average the velocity loss of the latest set to achieve the same purpose. However, given the ease of the use of RPE, it can be concluded that RPE is a more practical and global indirect fatigue marker during power BP.

To compare the validity of RPE and velocity loss as fatigue indicators not only after a specific set but also during the lifting process, the SFI, RPE, and velocity loss were examined throughout the exercise. Given that participants performed different numbers of repetitions across experimental conditions, the first, mid-point, and last repetitions were extracted for two-way ANOVA. Significant differences in the SFI were observed at the mid-point (L vs. H: *p* =0.014) and the last repetition (L vs. M: *p* =0.007; M vs. H: *p* =0.013; L vs. H: *p* <0.001). Similarly, significant differences in intra-set velocity loss were noted at the mid-point (L vs. M: *p* =0.005; L vs. H: *p* <0.001) and the last repetition (L vs. M: *p* <0.001; L vs. H: *p* <0.001). A significant correlation was also observed between the SFI and velocity loss, providing further evidence of the relationship between fatigue and velocity loss, consistent with previous studies that examined this relationship through blood lactate and ammonia concentrations 2 . In this study, using a highly sensitive sEMG-based algorithm, similar results were observed, confirming that the velocity loss is a reliable indicator of fatigue in upper-body power-aimed resistance exercises such as power BP. For RPE, significant differences were found at the mid-point (L vs. M: *p* =0.004; M vs. H: *p* <0.001; L vs. H: *p* <0.001) and the last repetition (all *p* <0.001). The significant correlation between the SFI and RPE supports and extends previous findings on the relationship between RPE and fatigue. Previous studies demonstrated that the RPE is a valid marker of fatigue in non-explosive resistance exercises, including non-explosive BP 14 35 36 . The present study shows that the RPE is also a reliable marker for indirect fatigue assessments in both non-explosive and power-aimed lifting settings. Based on the correlation comparison, no significant difference was found between the SFI–RPE and SFI-velocity loss correlation coefficients. Therefore, it can be concluded that both RPE and velocity loss effectively predict fatigue and can be used interchangeably as indirect fatigue indicators. Strength coaches and personal trainers can monitor RPE or velocity loss on a repetition-by-repetition basis or at predetermined points (e.g., the first, mid-point, and last repetition) to track fatigue during the lifting process. However, when using RPE-based fatigue assessments, there is no need for special devices or equipment (e.g., linear encoders), making the RPE a more practical option for assessing fatigue in power-aimed resistance exercise settings.

An interesting finding in this study was the significant correlation observed between velocity loss and RPE. Previous studies focusing on submaximal set configurations found a significant correlation between the OMNI scale (a resistance exercise-specific RPE scale) and mean concentric velocity during leg press exercises 26 . While these studies demonstrated that RPE could be used as a fatigue indicator, direct evidence linking RPE to fatigue was lacking. In the present study, we chose Borg’s CR-10 scale due to its broader applicability compared to the OMNI scale. We aimed to address the gap in previous research by assessing both velocity loss and fatigue. Our results successfully established a relationship between RPE, velocity loss, and fatigue. Consequently, it can be concluded that the RPE not only serves as a fatigue indicator but also reflects velocity decrease during power-aimed resistance exercises. In situations where velocity-assessing devices are unavailable, practitioners can use RPE as a simplified velocity indicator. By ceasing the set when RPE increases significantly, they can effectively avoid substantial velocity loss.

An important finding in this study was the lack of significant velocity loss during the L condition (the main effect for repetition: *p* =0.238). Previous studies advocating for velocity loss as a marker of fatigue did not establish clear limits for its application 2 26 . This finding introduces a new boundary for using velocity-based fatigue assessments, suggesting that velocity loss may not accurately reflect fatigue under certain power-aimed lifting conditions. For instance, significant velocity loss may not be observed when the prescribed volume is well below physical failure (e.g., ≤30% of the until-failure volume). In such cases, relying solely on velocity assessments could lead to an oversight of fatigue. This finding emphasizes the need for coaches and trainers to be cautious when using velocity-based fatigue assessments as the significant velocity loss is more likely to be absent when the volume settings are less than 30% of the until-failure volume. Additionally, velocity loss may offer limited precision in detecting subtle fatigue conditions. To enhance the accuracy of fatigue assessments in low-volume power-aimed training programs, it is advisable not to rely solely on velocity loss. Coaches and trainers should combine velocity loss with other fatigue-related markers, such as RPE and maximal countermovement jump height, to improve the precision of fatigue assessments, especially when fatigue may already be present despite an absence of the significant velocity decrease. This unexpected finding could also be interpreted in a reverse manner. As mentioned previously, practitioners should focus on avoiding significant velocity loss when performing power-aimed training programs. In the present study, significant velocity loss was not observed under the L condition. Accordingly, it could be concluded that lifting performance remains stable or decreases only slightly when the prescribed volume is low (e.g., ≤30% of the until-failure volume) with moderate relative intensity (e.g., 65% 1RM). Coaches and athletes could consider 30% of the until-failure volume as a threshold if their goal is to minimize performance loss during power-aimed resistance training programs. However, practitioners aiming to achieve both hypertrophy and power within a single training session, or applying velocity loss as an acute fatigue indicator during hypertrophy-aimed training programs, should exercise caution when using velocity loss as the sole fatigue indicator 53 . In such cases, velocity loss may provide misleading results, potentially underestimating the actual fatigue condition.

The present study has several limitations. First, to obtain intra-set RPE, a 2-second pause was included between repetitions. It is challenging to completely avoid the influence of this pause phase on fatigue, as it resembles isometric contraction, which may affect the results. Second, this study only assessed power BP exercises as it is a key exercise for developing upper-body power. However, these findings may not be directly applicable to other resistance exercises, particularly those involving the lower body. Previous research, along with our own studies, suggests that fatigue in the lower body is more complex due to physiological differences such as muscle fiber composition and lactate kinetics 36 54 . In a recently published work, velocity loss failed to increase even as the power back squat process approached physical failure 55 . Third, although the sample size was predetermined using statistical power analysis, it remains relatively small, which may have limited the statistical significance of some analyses. For instance, significant differences were not observed in certain pairwise comparisons. Future studies should aim to reduce the impact of the pause phase on intra-set data, directly compare the differences between upper- and lower-body power-aimed exercises, and involve a larger sample size to confirm the statistical power of the current findings.

## Conclusions

The present study demonstrated that the RPE, velocity loss, and SFI changed in a similar manner, indicating a strong relationship between subjective exertion, velocity decrease, and fatigue levels. Significant correlations were observed in SFI–RPE and SFI–velocity loss, leading us to conclude that both the RPE and velocity loss are valid indicators of fatigue during power BP exercises. Additionally, we found that the RPE is significantly correlated with velocity loss during power BP, suggesting that the RPE can serve as a simplified marker for velocity loss when velocity-measuring devices are unavailable in power-aimed resistance exercises. However, it is important to note that the velocity loss may not accurately reflect fatigue when the prescribed volume is low. Overall, regarding the similar changes and significant relationship between the RPE and the SFI, the RPE is a valid and practical indicator of fatigue, especially in power-aimed upper-body exercises.
